# Deciphering tea tree chloroplast and mitochondrial genomes of *Camellia sinensis* var. *assamica*

**DOI:** 10.1038/s41597-019-0201-8

**Published:** 2019-10-17

**Authors:** Fen Zhang, Wei Li, Cheng-wen Gao, Dan Zhang, Li-zhi Gao

**Affiliations:** 1 Institution of Genomics and Bioinformatics, South China Agricultural University, Guangzhou, 510642 China; 2 Affiliated Hospital, Qingdao University, Qingdao, 266003 China; 3 Plant Germplasm and Genomics Center, Germplasm Bank of Wild Species in Southwestern China, Kunming Institute of Botany, Chinese Academy of Sciences, Kunming, 650204 China

**Keywords:** Plant breeding, DNA sequencing, Genome, Sequence annotation

## Abstract

Tea is the most popular non-alcoholic caffeine-containing and the oldest beverage in the world. In this study, we *de novo* assembled the chloroplast (cp) and mitochondrial (mt) genomes of *C*. *sinensis* var. *assamica* cv. *Yunkang10* into a circular contig of 157,100 bp and two complete circular scaffolds (701719 bp and 177329 bp), respectively. We correspondingly annotated a total of 141 cp genes and 71 mt genes. Comparative analysis suggests repeat-rich nature of the mt genome compared to the cp genome, for example, with the characterization of 37,878 bp and 149 bp of long repeat sequences and 665 and 214 SSRs, respectively. We also detected 478 RNA-editing sites in 42 protein-coding mt genes, which are ~4.4-fold more than 54 RNA-editing sites detected in 21 protein-coding cp genes. The high-quality cp and mt genomes of *C*. *sinensis* var. *assamica* presented in this study will become an important resource for a range of genetic, functional, evolutionary and comparative genomic studies in tea tree and other *Camellia* species of the Theaceae family.

## Abstract (summary)

**Table:** 

Measurement(s)	genome assembly
Technology Type(s)	DNA sequencing
Sample Characteristic - Organism	Camellia sinensis

Machine-accessible metadata file describing the reported data: 10.6084/m9.figshare.9884729

## Background & Summary

Tea is the most popular non-alcoholic caffeine-containing and the oldest beverage in the world since 3000 B. C.^1,2^. The production of tea made from the young leaves of *Camellia sinensis* var. *sinensis* and *C*. *sinensis* var. *assamica*, together with ornamentally well-known camellias (e.g., *C*. *japonica*, *C*. *reticulata* and *C*. *sasanqua*) and worldwide renowned wooden oil crop *C*. *oleifera*^3^ has made the genus *Camellia* possess huge economic values in Theaceae. Besides its industrial, cultural and medicinal values, botanists and evolutionary biologists have increasingly paid attention to this genus. As a result of frequent hybridization and polyploidization, *Camellia* is almost commonly regarded as one of the most taxonomically and phylogenetically difficult taxa in flowering plants^4^. Thus, it has long been problematic for the taxonomic classification of the *Camellia* species based on the morphological characteristics^5^. The chloroplast (cp) genomes are able to provide valuable information for taxonomic classification, tracing source populations^6,7^ and the reconstruction of phylogeny to resolve complex evolutionary relationships^8–10^ due to the conservation of genomic structure, maternal inheritance and a fairly low recombination rate. Genetically speaking, cp genomes are comparatively conserved than plant mitochondria (mt) genomes which are more heterogeneous in nature. However, the presence of NUPT (nuclear plastid DNA) into cp genomes argues that cp genomes assembled from WGS data may include the heterogeneity due to the nuclear cp DNA transferred to the nucleus, resulting in erroneous phylogenetic inferences^11^. It has long been acknowledged that mtDNA has the propensity to integrate DNA from various sources through intracellular and horizontal transfer^12–14^. Partially due to these reasons, the mt genomes vary from ~200 Kbp to ~11.3 Mbp in some living organisms^15–17^. The dynamic nature of mt genome structure has been recognized, and plant mt genomes can have a variety of different genomic configurations due to the recombination and differences in repeat content^18,19^. These characteristics make the plant mt genome a fascinating genetic system to investigate questions related to evolutionary biology. The first effort has been made to sequence the 13 representative *Camellia* chloroplast genomes using next-generation Illumina genome sequencing platform, which obtained novel insights into global patterns of structural variation across the *Camellia* cp genomes^4^. The reconstruction of phylogenetic relationships among these representative species of *Camellia* suggests that cp genomic resources are able to provide useful data to help to understand their evolutionary relationships and classify the ‘difficult taxa’. Increasing interest in the *Camellia* plants have made up to thirty-eight of cp genomes be sequenced up to date^20–37^. Recently, we decoded the first nuclear genome of *C*. *sinensis* var. *assamica* cv. *Yunkang10*, providing novel insights into genomic basis of tea flavors^38^. Besides the lack of the *C*. *sinensis* var. *assamica* cp genome among thirty-eight cp genomes that were sequenced in this genus^4,20–37^, up to data, none of mt genome has been determined in the genus *Camellia*.

In this study, we filtered cpDNA and mtDNA reads from the WGS genome sequence project^38^ and *de novo* assembled the mt genome and cp genome of *C*. *sinensis* var. *assamica*. The information of both cp and mt genomes will help to obtain a comprehensive understanding of the taxonomy and evolution of the genus *Camellia*. These genome sequences will also facilitate the genetic modification of these economically important plants, for example, through chloroplast genetic engineering technologies.

## Methods

### Plant materials, DNA extraction and genome sequencing

Young and healthy leaves of an individual plant of cultivar *Yunkang10* of *C*. *sinensis* var. *assamica* were collected for genome sequencing in April, 2009, from Menghai County, Yunnan Province, China. Fresh leaves were harvested and immediately frozen in liquid nitrogen after collection, followed by the preservation at −80 °C in the laboratory prior to DNA extraction. High-quality genomic DNA was extracted from leaves using a modified CTAB method^39^. RNase A and proteinase K were separately used to remove RNA and protein contamination. The quality and quantity of the isolated DNA were separately checked by electrophoresis on a 0.8% agarose gel and a NanoDrop D-1000 spectrophotometer (NanoDrop Technologies, Wilmington, DE). A total of eleven paired-end libraries, including four types of small-insert libraries (180 bp, 260 bp, 300 bp, 500 bp) and seven large-insert libraries (2 Kb, 3 Kb, 4 Kb, 5 Kb, 6 Kb, 8 Kb, 20 Kb), were prepared following the Illumina’s instructions, and sequenced using Illumina HiSeq. 2000 platform by following the standard Illumina protocols (Illumina, San Diego, CA). We totally generated ~707.88 Gb (~229.31×) of raw sequencing data^38^. Further reads quality control filtering processes yielded a total of ~492.15 Gb (~159.43×) high-quality data retained and used for subsequent genome assembly.

### *De novo* chloroplast and mitochondria genome assemblies

**Fig. 1:**
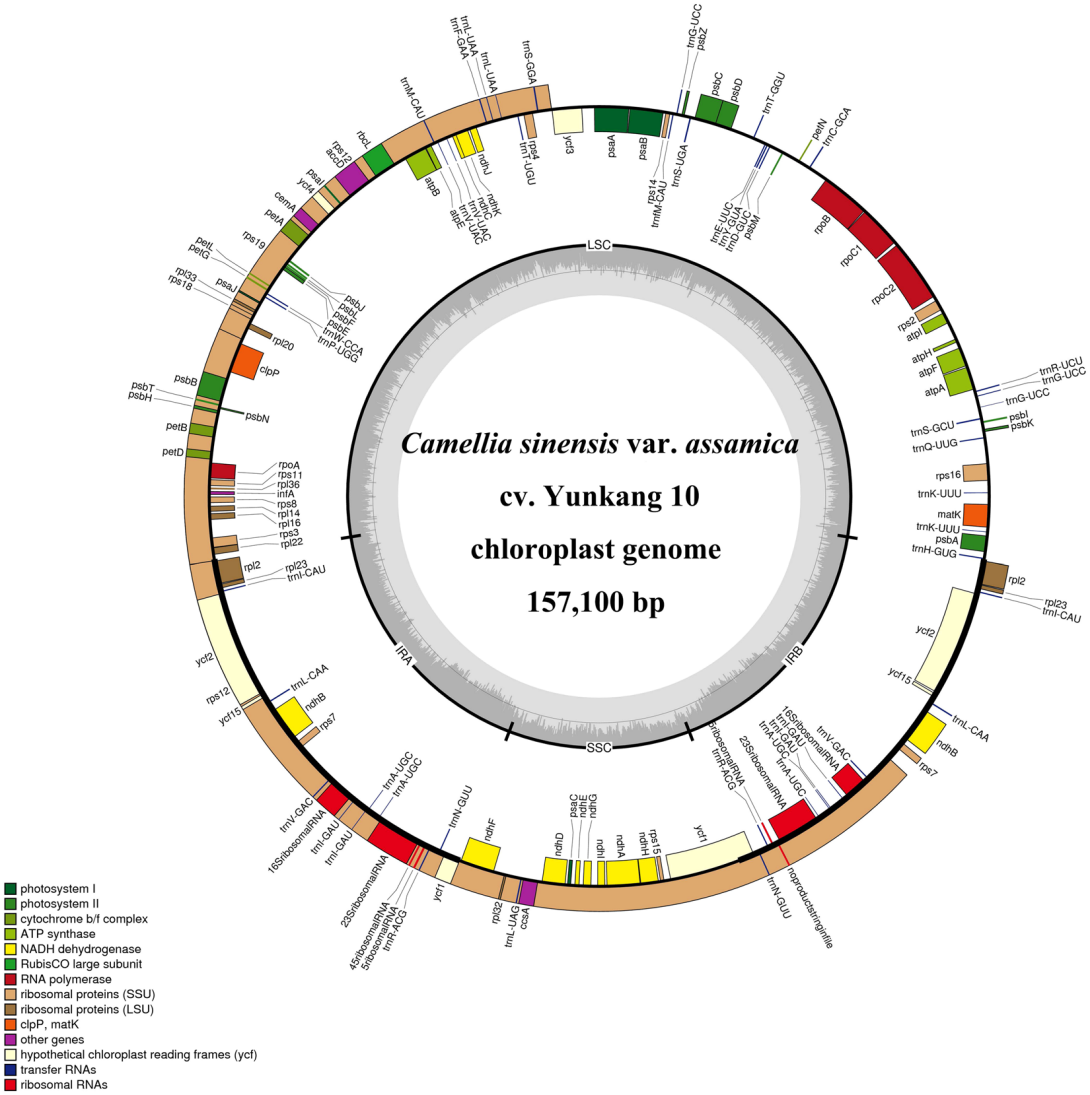
Genome map of *C*. *sinensis* var. *assamica* cv. *Yunkang10*. Genes lying outside of the outer circle are transcribed in the clockwise direction whereas genes inside are transcribed in the counterclockwise direction. Genes belonging to different functional groups are color-coded. Area dashed darker gray in the inner circle indicates GC content while the lighter gray corresponds to AT content of the genome.

**Fig. 2:**
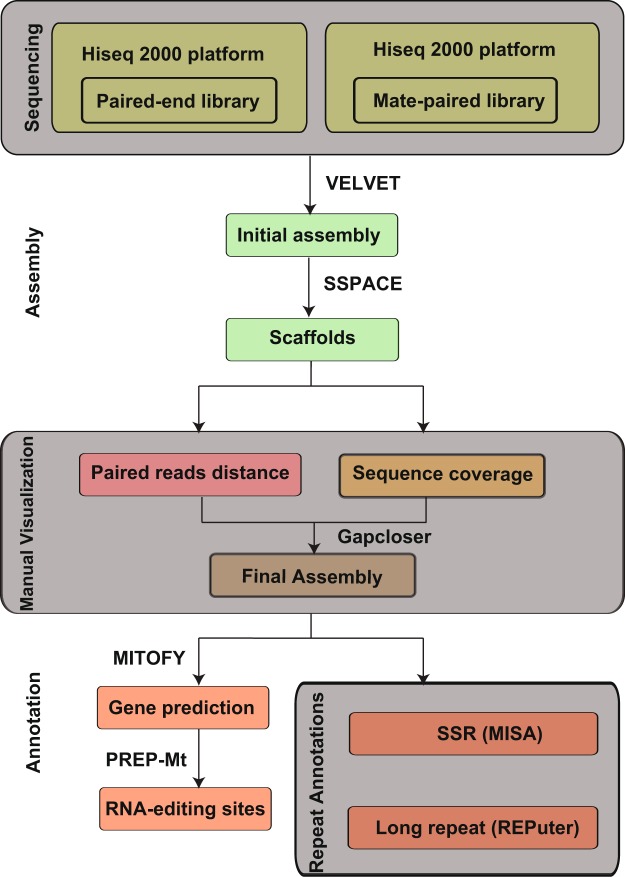
The assembly and annotation pipeline of the tea tree mitochondrial genome.

**Fig. 3:**
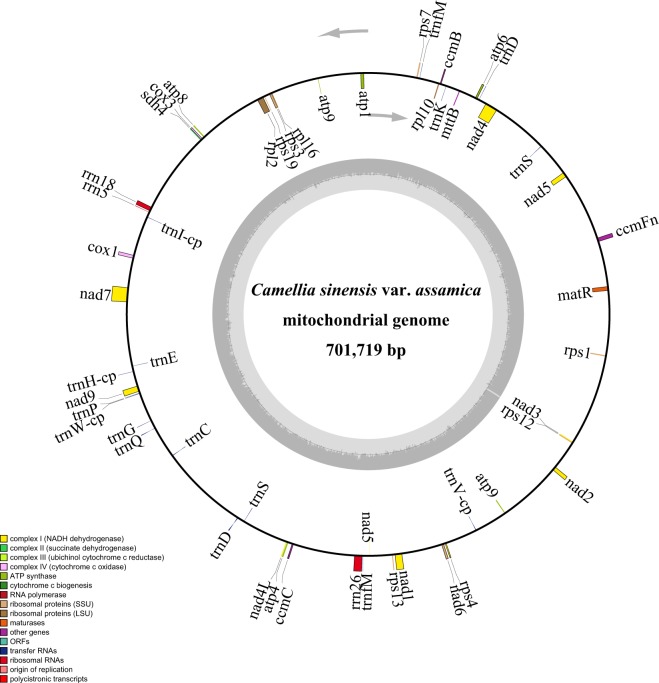
Circular map of scaffold 1 in the *C*. *sinensis* var. *assamica* cv. *Yunkang10* mitochondrial genome. Gene map showing 54 annotated genes with different functional groups that are color-coded on outer circle as transcribed clock-wise (outside) and transcribed counter clock-wise (inside). The inner circle indicates the GC content as dark grey plot.

**Fig. 4:**
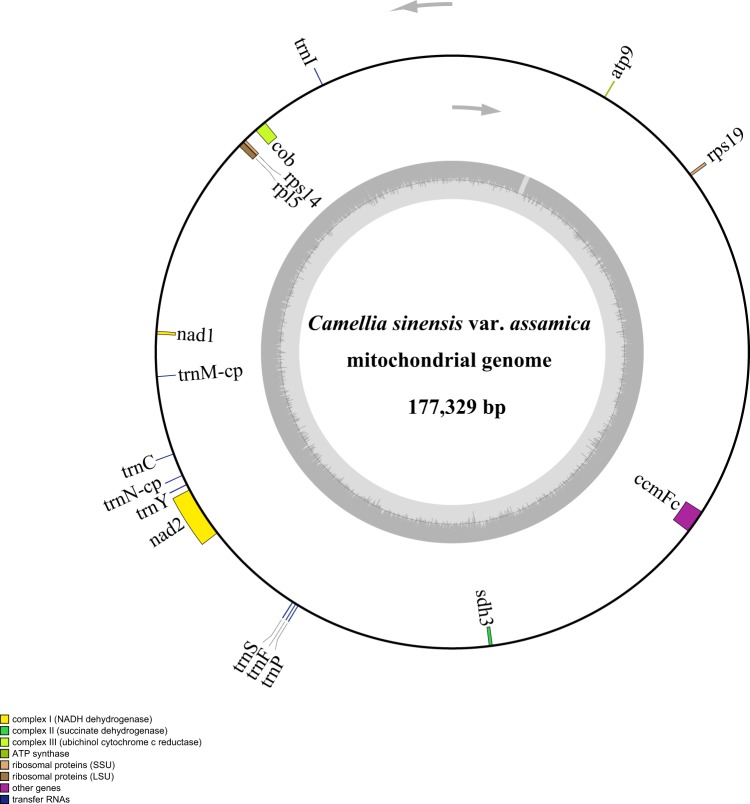
Circular map of scaffold 2 in the *C*. *sinensis* var. *assamica* cv. *Yunkang10* mitochondrial genome. Gene map showing 17 annotated genes with different functional groups that are color-coded on outer circle as transcribed clock-wise (outside) and transcribed counter clock-wise (inside). The inner circle indicates the GC content as dark grey plot.

The chloroplast reads were filtered from whole genome Illumina sequencing data of *C*. *sinensis* var. *assamica*, we mapped all the sequencing reads to the reference genomes^4^ using bowtie2 (version 2.3.4.3)^40^. The mapped chloroplast reads were assembled into a circular contig of 157,100 bp in length with an overall GC content of 37.29% using CLC Genomics Workbench v. 3.6.1 (CLC Inc., Rarhus, Denmark) (Fig. 1). For mitochondria genome assembly, the PE and MP sequencing reads were used separately. Briefly, we first performed *de novo* assembly with VELVET v1.2.08^41^, which was previously described^42,43^. Scaffolds were constructed using SSPACE v.3.0^44^. False connection was manually removed based on the coverage and distances of paired reads. Gaps between scaffolds were then filled with GapCloser (version 1.12)^45,46^ using all pair-end reads. We obtained the two complete circular scaffolds (701719 bp and 177329 bp) of the *C*. *sinensis* var. *assamica* mt genome from the *de-novo* assembly of the filtered mitochondrial reads (Figs 2–4**)**. The two scaffolds of the mt genome had overall GC contents of 45.63% and 45.81%, respectively. The completed chloroplast and mitochondria genomes are publicly available in NCBI GenBank under accession numbers MH019307, MK574876 and MK574877 and BIG Genome Warehouse WGS000271, WGS000272.

### Genome annotation and visualization

The complete chloroplast genome of *C*. *sinensis* var. *assamica* was preliminarily annotated using the online program DOGMA^47^ (Dual Organellar Genome Annotator) followed by manual correction. A total of 141 genes were annotated, of which 87 were protein-coding genes, 46 were tRNA genes and eight were rRNA genes **(**Table 1**)**. MITOFY^15^ was used to characterize the complement of protein-coding and rRNA genes in the mitochondrial genome. A tRNA gene search was carried out using the tRNA scan-SE software (version 1.3.1)^48^. We annotated a total of 71 genes, including 44 protein-coding genes, 24 tRNAs and 3 rRNAs (Table 2). Circular genome maps were drawn with OrganellarGenomeDRAW^49^ (Figs 3–4**)**.

**Table 1:** Gene annotation of the *C*. *sinensis* var. *assamica* cp genome.

Category	Group	Genes
Photosynthesis related genes	Rubisco	rbcL
	Photosystem I	psaA, psaB, psaC, psaI, psaJ
	Assembly/stability of Photosystem I	ycf3
	Photosystem II	psbA, psbB, psbT, psbK, psbI, psbH, psbM, psbN, psbD, psbC, psbZ, psbJ, psbL, psbE, psbF
	ATP synthase	atpA, atpB, atpE, atpF, atpH, atpI
	Cytochrome b/f complex	petA, petB, petD, petN, petL, petG
	Cytochrome csynthesis	ccsA
	NADPH dehydrogenase	ndhA, ndhB (×2), ndhC, ndhD, ndhE, ndhF, ndhH, ndhG, ndhJ, ndhK, ndhI
Transcription and translation related genes	Transcription	rpoA, rpoC2, rpoC1, rpoB
	Ribosomal proteins	rps2, rps3, rps4, rps7 (×2), rps8, rps11, rps12, rps14, rps15, rps16, rps18, rps19, rpl2 (×2), rpl14, rpl16, rpl20, rpl22, rpl23 (×2), rpl32, rpl33, rpl36
	Translation initiation factor	infA
RNA genes	Ribosomal RNA	rrn16S (×2), rrn23S (×2), rrn4.5 (×2), rrn5 (×2)
	Transfer RNA	trnH-GUG, trnK-UUU (×2), trnQ-UUG, trnS-GCU, trnG-UCC (×2), trnR-UCU, trnC-GCA, trnD-GUC, trnY-GUA, trnE-UUC, trnT-GGU, trnS-UGA, trnG-UCC, trnfM-CAU, trnS-GGA, trnT-UGU, trnL-UAA (×2), trnF-GAA, trnV-UAC (×2), trnM-CAU, trnW-CCA, trnP-UGG, trnI-CAU, trnL-CAA (×2), trnV-GAC, trnI-GAU (×3), trnA-UGC (×2), trnR-ACG (×2), trnN-GUU (×2), trnL-UAG, trnN-GUU, trnR-ACG, trnA-UGC (×2), trnV-GAC, trnI-CAU
Other genes	RNA processing	matK
	Carbon metabolism	cemA
	Fatty acid synthesis	accD
	Proteolysis	clpP
Genes of unknown function	Conserved ORFs	ycf1 (×2), cf2, ycf4, ycf2, ycf15 (×2)

**Table 2:** Gene content of the *C*. *sinensis* var. *assamica* mt genome.

Group of genes	Name of genes	
	Scaffold 1	Scaffold 2
Complex I	nad1, nad2, nad3, nad4, nad4L, nad5, nad6, nad7, nad9 (×2)	nad1, nad2
Complex II	sdh3, sdh4	sdh3
Complex III		cob
Complex IV	cox1, cox2, cox3	
Complex V	atp1, atp4, atp6, atp8, atp9	atp9
Cytochrome c biogenesis	ccmFn, ccmB, ccmC	ccmFc
Ribosome large subunit	rpl2, rpl10, rpl16	rpl5
Ribosome small subunit	rps1, rps3, rps4, rps7, rps12, rps13, rps19	rps14, rps19
rRNA genes	rrn5, rrn18, rrn16	
tRNA genes	trnS(Ser), trnD(Asp), trnK(Lys), trnfM(Met) (×2), trnI(Ile)-cp, trnE(Glu), trnH(His)-cp, trnP(Pro), trnW(Trp)-cp, trnG(Gly), trnQ(Gln), trnC(Cys), trnD(Asp), trnS(Ser), trnV(Val)-cp	trnI(Ile), trnM(Met)-cp, trnC(Cys), trnN(Asn)-cp, trnY(Tyr), trnS(Ser), trnF(Phe), trnP(Pro)
chloroplast-derived genes	trnI(Ile)-cp, trnH(His)-cp, trnW(Trp)-cp, trnV(Val)-cp	trnM(Met)-cp, trnN(Asn)-cp
Other proteins	matR, mttB	

Simple sequence repeats (SSRs) were identified and located using MISA (http://pgrc.ipk-gatersleben.de/misa/). All the annotated SSRs were classified by the size and copy number of their tandemly repeated: monomer (one nucleotide, n ≥ 8), dimer (two nucleotides, n ≥ 4), trimer (three nucleotides, n ≥ 4), tetramer (four nucleotides, n ≥ 3), pentamer (five nucleotides, n ≥ 3), hexamer (six nucleotides, n ≥ 3). A total of 214 SSRs were identified in cp genome with 74.42% of which were monomers, 19.07% of dimers, 0.47% of trimers, 4.65% of tetramers and 0.93% of hexamers (Table 3). There were no pentamers found in the cp genome. In mt genome, we obtained 665 SSRs distributed into monomers, dimers, trimers, pentamers, tetramers and hexamers with 31.53%, 45.35%, 4.95%, 15.17%, 2.70% and 0.15%, respectively (Table 3). Repeat sequences including forward and palindromic repeats, were also searched by REPuter^50^ with the following parameters: minimal length 50 nt; mismatch 3 nt. Long repeat sequences (repeat unit > 50 bp) of forward and palindromic repeats were further annotated, resulting in 149 bp from 4 paired repeats in the cp genome (Table 4) and 37,878 bp from 58 paired repeats in the mt genome (Online-only Tables 1–2). Our repeat content analyses indicate that the mt genome is more abundant in repeat sequences and more variable than the cp genome of *C*. *sinensis* var. *assamica* (Table 4**;** Online-only Tables 1–2**)**.

**Table 3:** Statistics of SSR motifs in the *C*. *sinensis* var. *assamica* mt and cp genomes.

SSR-Motif	mt Genome		cp Genome	
	SSR Number	SSR %	SSR Number	SSR %
Monomer	210	31.53	160	74.42
Dimer	302	45.35	41	19.07
Trimer	33	4.95	1	0.47
Tetramer	101	15.17	10	4.65
Pentamer	18	2.70	0	0.00
Hexamer	1	0.15	2	0.93

**Table 4:** Long repeats (repeat unit > 50 bp) in the *C*. *sinensis* var. *assamica* cp genome.

Repeat Length	Type*	Start of Copy 1	Start of Copy 2
56	F	93938	93956
56	P	93938	149737
56	P	93956	149755
56	F	149737	149755

*P indicates palindromic repeats; F indicates forward repeats.

Overlapped repeats have been manually removed while calculating total length.

### Prediction of RNA-editing sites

Putative RNA editing sites in protein-coding genes were predicted using the PREP-cp and PREP-mt Web-based program (http://prep.unl.edu/)^51,52^. To achieve a balanced trade-off between the number of false positive and false negative sites, the cutoff score (C-value) was set to 0.8 and 0.6, respectively^53^.

Almost all transcripts of protein encoding genes in the plant mitochondria are subject to RNA editing except the *T-urf13* gene^54^. Our results showed that the extent of RNA editing varied by gene for both cp and mt genomes of *C*. *sinensis* var. *assamica*. In the *C*. *sinensis* var. *assamica* cp genome, we detected 54 RNA-editing sites in 21 protein-coding genes, ranging from one editing site in *atpF*, *atpI*, *petB*, *psaI*, *psbE*, *psbF*, *rpoA*, *rps2* and *rps8* to 8 editing sites in *ndhB* (Online-only Table 3). In the *C*. *sinensis* var. *assamica* mt genome, we predicted 478 RNA-editing sites in 42 protein-coding genes; they varied from two editing site in *atp9* (of scaffold2), *sdh3* (of scaffold1 and scaffold2, respectively) and *rps14* (of scaffold2) to 35 editing sites in *ccmFn* (of scaffold1) (Online-only Table 4–5).

### Phylogenetic analyses

**Fig. 5:**
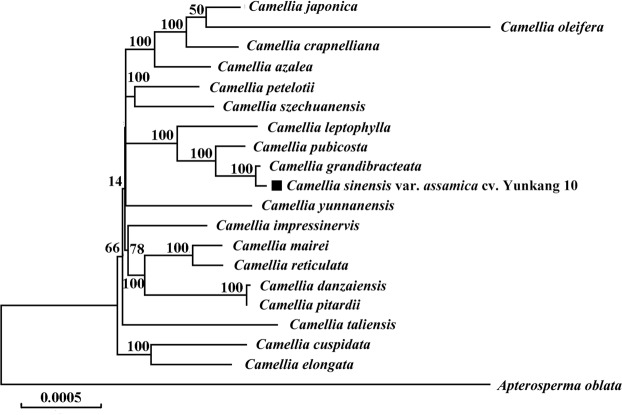
Phylogenetic relationships of 20 complete chloroplast genomes. Maximum likelihood phylogenetic tree of *C*. *sinensis* var. *assamica* cv. *Yunkang 10* with 18 species in the genus *Camellia* based on complete chloroplast genome sequences. The chloroplast sequence of *Apterosperma oblata* was set as outgroup. The position of *C*. *sinensis* var. *assamica* cv. *Yunkang 10* is shown in bold and bootstrap values are shown for each node.

To further determine the phylogenetic position of *C*. *sinensis* var. *assamica* we performed phylogenomic analysis of 20 complete cp genomes using the GTR + R + I model under the maximum likelihood (ML) inference in MEGA v.7.0^55^. Besides *C*. *sinensis* var. *assamica* cv. *Yunkang 10*, we selected cp genomes from the eighteen *Camelia* species (*C*. *oleifera*, *C*. *crapnelliana*, *C*. *szechuanensis*, *C*. *mairei*, *C*. *elongata*, *C*. *grandibracteata*, *C*. *leptophylla*, *C*. *petelotii*, *C*. *pubicosta*, *C*. *reticulata*, *C*. *azalea*, *C*. *japonica*, *C*. *cuspidata*, *C*. *danzaiensis*, *C*. *impressinervis*, *C*. *pitardii*, *C*. *yunnanensis* and *C*. *taliensis*) using *Apterosperm oblata* as outgroup. Our results showed that *C*. *sinensis* var. *assamica* was grouped with *C*. *grandibracteata* with 100% bootstrap support (Fig. 5).

**Fig. 6:**
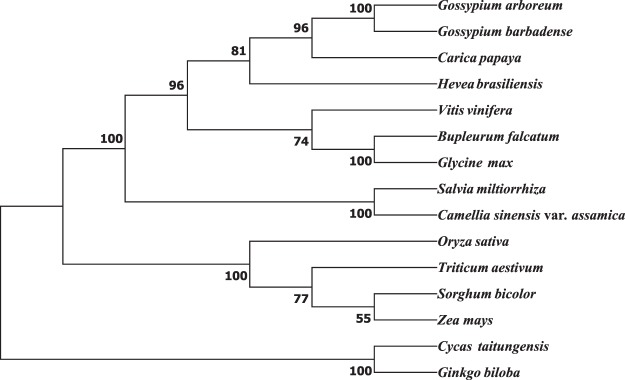
Phylogeny inferred from 13 genes common in the 15 plant mitochondrial genomes. Neighbor-joining tree of *C*. *sinensis* var. *assamica* cv. *Yunkang 10* with other 14 species based on 13 conserved protein-coding gene sequences with bootstrap support values on each node. The mt sequence of *Cycas taitungensis* and *Ginkgo biloba* were set as outgroup.

The same method was used for phylogenetic analysis with mt genome. A total of thirteen conserved mt protein-coding genes among *C*. *sinensis* var. *assamica* and 14 other plant species were individually aligned with ClustalW^56^, and then concatenated to construct a contiguous sequence in the order of *cob*, *cox1*, *cox2*, *cox3*, *nad1*, *nad2*, *nad3*, *nad4*, *nad4L*, *nad5*, *nad6*, *nad7* and *nad9*. The selected 14 species includes *Cycas taitungensis*, *Ginkgo biloba*, *Triticum aestivum*, *Oryza sativa*, *Sorghum bicolor*, *Zea mays*, *Gossypium arboretum*, *G*. *barbadense*, *Carica papaya*, *Vitis vinifera*, *Hevea brasiliensis*, *Bupleurum falcatum*, *Glycine max* and *Salvia miltiorrhiza*. The alignment file was used for the construction of Neighbor-Joining Tree at 1000 bootstrap replicates with MEGA 7.0.26^55^. Our results showed that *C*. *sinensis* var. *assamica* is clearly grouped with other dicots that were separated from monocots of the angiosperms while the two gymnosperms (*Cycas taitungensis* and *Ginkgo biloba*) were formed the basal clade (Fig. 6).

## Data Records

Raw reads from Illumina are deposited in the NCBI Sequence Read Archive (SRA)^57–62^ and BIG Genome Warehouse^63^. Assembled cp genome sequences and accompanying gene annotations of *C*. *sinensis* var. *assamica* are deposited in the NCBI GenBank^64^ and BIG Genome Warehouse^65^. The mt genome final assembly and accompanying gene annotations are deposited at NCBI GenBank^66,67^ and BIG Genome Warehouse^68^. The alignment and tree files of the chloroplast genome and mitochondrial genome form the Camellia genus were deposited in Figshare database^69^.

## Technical Validation

### Quality filtering of raw reads

The initially generated raw sequencing reads were evaluated in terms of the average quality score at each position, GC content distribution, quality distribution, base composition, and other metrics. Furthermore, the sequencing reads with low quality were also filtered out before the genome assembly and annotation of gene structure.

### Assembly and validation

The chloroplast reads were filtered from whole genome Illumina sequencing data of *C*. *sinensis* var. *assamica*. We mapped all the cleaned reads to the reference chloroplast sequence^4^ using bowtie2 (version 2.3.4.3)^40^ with default parameters. The mapped chloroplast reads were *de novo* assembled into the complete chloroplast genome.

For mitochondria genome assembly, the PE and MP sequencing reads were used separately. Briefly, we first performed *de novo* assembly with VELVET v1.2.08^41^, which was previously described^42,43^. Scaffolds were constructed using SSPACE v.3.0^44^. False connection was manually removed based on the coverage and distances of paired reads. Gaps between scaffolds were then filled with GapCloser (version 1.12)^45,46^ using all pair-end reads.

## Data Availability

The following bioinformatic tools and versions were used for generating all results as described in the main text: 1. Bowtie2, version 2.3.4.3, was used for aligning sequencing reads to long reference sequences with default parameters: http://bowtie-bio.sourceforge.net/bowtie2/index.shtml 2. CLC Genomics Workbench, version 3.6.1, was used for genome assembly with default parameters: https://www.qiagenbioinformatics.com/products/clc-genomics-workbench/ 3. Velvet, version 1.2.08, was used for genome *de novo* assembly, which was previously described: https://www.ebi.ac.uk/~zerbino/velvet/ 4. SSPACE, version 3.0, was used for genome scaffolds assembly with default parameters: https://www.baseclear.com/services/bioinformatics/basetools/sspace-standard/ 5. GapCloser, version 1.12, was used to fill the gaps between scaffolds with default parameters: https://sourceforge.net/projects/soapdenovo2/files/GapCloser/ 6. DOGMA (an online tool), accessed at 12/2018, was used for annotating cp genomes with default parameters: http://dogma.ccbb.utexas.edu/ 7. Mitofy (an online tool), accessed at 12/2018, was used for annotating plant mt genomes with default parameters: http://dogma.ccbb.utexas.edu/mitofy/ 8. tRNAscanSE, VERSION 1.3.1, was used to search tRNA with default parameters: http://lowelab.ucsc.edu/tRNAscan-SE/ 9. Organellar Genome DRAW (an online tool), accessed at 12/2018, was used for creating high quality visual representation of cp gemome with default parameters: https://chlorobox.mpimp-golm.mpg.de/OGDraw.html 10. MISA,version 1.0, was used for annotating SSR with monomer (one nucleotide, n ≥ 8), dimer (two nucleotides, n ≥ 4), trimer (three nucleotides, n ≥ 4), tetramer (four nucleotides, n ≥ 3), pentamer (five nucleotides, n ≥ 3), hexamer (six nucleotides, n ≥ 3): http://pgrc.ipk-gatersleben.de/misa/misa.html 11. REPuter (an online tool), accessed at 1/2019, was used for annotating long repeated sequences with the following parameters: minimal length 50 nt; mis match 3 nt: https://bibiserv.cebitec.uni-bielefeld.de/reputer/ 12. PREP-cp (an online tool), accessed at 1/2019, was used for predicting RNA editor for plant cp genes with the cutoff score (C-value) setting to 0.8: http://prep.unl.edu/ 13. PREP-mt (an online tool), accessed at 1/2019, was used for predicting RNA editor for plant mt genes with the cutoff score (C-value) setting to 0.6: http://prep.unl.edu/ 14. MEGA, version 7.0.26, was used for phylogenomics and phylomedicine at 1000 bootstrap: https://www.megasoftware.net/ 15. ClustalW, version 2, was used for multiple sequence alignment with default parameters: https://www.ebi.ac.uk/Tools/msa/clustalw2/

## Online-only Tables

**Online-only Table 1:** Long repeats (repeat unit > 50 bp) in Scaffold 1 of the *C*. *sinensis* var. *assamica* mt genome.

Repeat Length	Type*	Start of Copy 2	Start of Copy 1
5119	F	207173	443366
2191	F	389017	391244
1963	F	210330	212292
1962	F	212292	446523
1930	F	383226	385188
1650	F	205522	207173
1650	F	205522	443366
1469	F	538290	539780
814	F	496567	498047
705	F	619432	621461
665	F	497382	498862
255	P	151984	200526
228	P	448476	544136
204	F	277002	363807
131	P	73675	482324
125	F	301855	468834
104	F	297204	623713
88	F	228824	559689
87	F	594334	641398
84	F	530415	646532
82	P	224027	395044
82	F	509347	623862
81	P	152363	200041
80	F	304361	306020
78	P	299987	587603
74	F	165777	570981
70	F	165878	571083
69	F	123050	384677
69	F	123050	386639
67	F	18495	27472
66	F	299782	537227
66	P	364849	599005
66	F	684228	684285
65	P	508609	683320
64	F	542385	560020
63	F	605770	619261
62	P	70098	424512
62	F	151516	524252
62	P	156839	486845
61	F	123120	384747
61	F	123120	386709
61	P	142673	486240
60	F	302012	395122
59	P	265260	472040
58	F	285626	402303
57	P	152478	199950
57	F	276881	363698
56	F	402376	658389
55	P	41703	667438
55	F	258578	486959

*P indicates palindromic repeats; F indicates forward repeats. Overlapped repeats have been manually removed while calculating total length.

**Online-only Table 2:** Long repeats (repeat unit > 50 bp) in Scaffold 2 of the *C*. *sinensis* var. *assamica* mt genome.

Repeat Length	Type*	Start of Copy 1	Start of Copy 2
704	F	30739	32294
156	P	29085	67620
86	F	67291	136332
67	P	4255	17574
67	P	23998	45730
62	F	67282	135282
55	F	120664	129253
53	F	135291	136332

*P indicates palindromic repeats; F indicates forward repeats. Overlapped repeats have been manually removed while calculating total length.

**Online-only Table 3:** Predicted RNA-editing sites in the *C*. *sinensis* var. *assamica* cp genome. (*The cutoff score (C-value) was set to 0.8).

No.	Gene	Nucleotide Pos	AA Pos	Effect	Score*
1	*accD*	64	22	CGG (R) => TGG (W)	1
2	*accD*	1469	490	CCT (P) => CTT (L)	1
3	*atpA*	791	264	CCA (P) => CTA (L)	1
4	*atpA*	914	305	TCA (S) => TTA (L)	1
5	*atpF*	92	31	CCA (P) => CTA (L)	0.86
6	*atpI*	134	45	GCT (A) => GTT (V)	1
7	*matK*	445	149	CAC (H) => TAC (Y)	1
8	*matK*	467	156	TCG (S) => TTG (L)	1
9	*matK*	631	211	CAT (H) => TAT (Y)	1
10	*matK*	1234	412	CAT (H) => TAT (Y)	1
11	*ndhA*	341	114	TCA (S) => TTA (L)	1
12	*ndhA*	566	189	TCA (S) => TTA (L)	1
13	*ndhA*	1028	343	TCT (S) => TTT (F)	1
14	*ndhA*	1073	358	TCT (S) => TTT (F)	1
15	*ndhB*	149	50	TCA (S) => TTA (L)	1
16	*ndhB*	467	156	CCA (P) => CTA (L)	1
17	*ndhB*	586	196	CAT (H) => TAT (Y)	1
18	*ndhB*	611	204	TCA (S) => TTA (L)	0.8
19	*ndhB*	737	246	CCA (P) => CTA (L)	1
20	*ndhB*	746	249	TCT (S) => TTT (F)	1
21	*ndhB*	830	277	TCA (S) => TTA (L)	1
22	*ndhB*	1481	494	CCA (P) => CTA (L)	1
23	*ndhD*	20	7	ACG (T) => ATG (M)	1
24	*ndhD*	401	134	TCA (S) => TTA (L)	1
25	*ndhD*	692	231	TCA (S) => TTA (L)	1
26	*ndhD*	896	299	TCA (S) => TTA (L)	1
27	*ndhD*	905	302	CCT (P) => CTT (L)	1
28	*ndhD*	1328	443	TCA (S) => TTA (L)	0.8
29	*ndhF*	205	69	CAT (H) => TAT (Y)	0.8
30	*ndhF*	290	97	TCA (S) => TTA (L)	1
31	*ndhG*	166	56	CAT (H) => TAT (Y)	0.8
32	*ndhG*	314	105	ACA (T) => ATA (I)	0.8
33	*petB*	641	214	CCA (P) => CTA (L)	1
34	*psaI*	80	27	TCT (S) => TTT (F)	0.86
35	*psbE*	214	72	CCT (P) => TCT (S)	1
36	*psbF*	77	26	TCT (S) => TTT (F)	1
37	*rpoA*	368	123	TCG (S) => TTG (L)	1
38	*rpoB*	338	113	TCT (S) => TTT (F)	1
39	*rpoB*	473	158	TCA (S) => TTA (L)	0.86
40	*rpoB*	551	184	TCA (S) => TTA (L)	1
41	*rpoB*	566	189	TCG (S) => TTG (L)	1
42	*rpoB*	973	325	CTT (L) => TTT (F)	0.86
43	*rpoB*	2000	667	TCT (S) => TTT (F)	1
44	*rpoB*	2336	779	ACA (T) => ATA (I)	1
45	*rpoC1*	41	14	TCA (S) => TTA (L)	1
46	*rpoC1*	1556	519	TCG (S) => TTG (L)	1
47	*rpoC2*	1505	502	ACG (T) => ATG (M)	0.86
48	*rpoC2*	2290	764	CGG (R) => TGG (W)	1
49	*rpoC2*	2726	909	ACT (T) => ATT (I)	1
50	*rpoC2*	3728	1243	TCA (S) => TTA (L)	0.86
51	*rps2*	248	83	TCA (S) => TTA (L)	1
52	*rps8*	182	61	TCA (S) => TTA (L)	0.86
53	*rps14*	80	27	TCA (S) => TTA (L)	1
54	*rps14*	149	50	CCA (P) => CTA (L)	1

**Online-only Table 4:** Predicted RNA-editing sites in Scaffold 1 of the *C*. *sinensis* var. *assamica* mt genome.

No.	Gene	Nucleotide Position	AA Pos	Effect	Score*
1	*matR*	32	11	TCC (S) => TTC (F)	0.62
2	*matR*	236	79	TCC (S) => TTC (F)	0.62
3	*matR*	326	109	CCA (P) => CTA (L)	1
4	*matR*	917	306	TCA (S) => TTA (L)	1
5	*matR*	1442	481	GCC (A) => GTC (V)	0.62
6	*matR*	1667	556	TCC (S) => TTC (F)	1
7	*matR*	1688	563	CCT (P) => CTT (L)	1
8	*matR*	1708	570	CGC (R) => TGC (C)	1
9	*matR*	1744	582	CAC (H) => TAC (Y)	1
10	*matR*	1775	592	CCG (P) => CTG (L)	1
11	*matR*	1814	605	CCA (P) => CTA (L)	0.88
12	*matR*	1832	611	TCA (S) => TTA (L)	0.88
13	*ccmFn*	38	13	CCG (P) => CTG (L)	1
14	*ccmFn*	98	33	CCT (P) => CTT (L)	1
15	*ccmFn*	137	46	TCG (S) => TTG (L)	1
16	*ccmFn*	142	48	CGT (R) => TGT (C)	1
17	*ccmFn*	151	51	CCT (P) => TCT (S)	0.83
18	*ccmFn*	248	83	TCA (S) => TTA (L)	1
19	*ccmFn*	256	86	CGG (R) => TGG (W)	1
20	*ccmFn*	283	95	CTT (L) => TTT (F)	0.83
21	*ccmFn*	334	112	CAT (H) => TAT (Y)	0.67
22	*ccmFn*	356	119	TCC (S) => TTC (F)	0.67
23	*ccmFn*	391	131	CCT (P) => TCT (S)	1
24	*ccmFn*	478	160	CGT (R) => TGT (C)	0.83
25	*ccmFn*	706	236	CCT (P) => TTT (F)	0.67
26	*ccmFn*	707	236	CCT (P) => TTT (F)	0.67
27	*ccmFn*	716	239	TCA (S) => TTA (L)	0.83
28	*ccmFn*	754	252	CGT (R) => TGT (C)	1
29	*ccmFn*	776	259	TCA (S) => TTA (L)	1
30	*ccmFn*	788	263	CCA (P) => CTA (L)	1
31	*ccmFn*	803	268	TCA (S) => TTA (L)	1
32	*ccmFn*	893	298	GCG (A) => GTG (V)	1
33	*ccmFn*	952	318	CGC (R) => TGC (C)	1
34	*ccmFn*	1270	424	CGG (R) => TGG (W)	1
35	*ccmFn*	1298	433	CCA (P) => CTA (L)	1
36	*ccmFn*	1315	439	CAT (H) => TAT (Y)	1
37	*ccmFn*	1330	444	CGG (R) => TGG (W)	1
38	*ccmFn*	1348	450	CGG (R) => TGG (W)	1
39	*ccmFn*	1381	461	CGG (R) => TGG (W)	1
40	*ccmFn*	1399	467	CGT (R) => TGT (C)	1
41	*ccmFn*	1442	481	TCG (S) => TTG (L)	1
42	*ccmFn*	1462	488	CTT (L) => TTT (F)	1
43	*ccmFn*	1466	489	CCA (P) => CTA (L)	1
44	*ccmFn*	1478	493	TCA (S) => TTA (L)	1
45	*ccmFn*	1487	496	TCT (S) => TTT (F)	1
46	*ccmFn*	1513	505	CCC (P) => TCC (S)	1
47	*ccmFn*	1561	521	CGG (R) => TGG (W)	0.67
48	*nad5*	155	52	CCG (P) => CTG (L)	1
49	*nad5*	238	80	CCG (P) => TCG (S)	0.8
50	*nad5*	269	90	TCC (S) => TTC (F)	0.7
51	*nad5*	355	119	CCT (P) => TTT (F)	1
52	*nad5*	356	119	CCT (P) => TTT (F)	1
53	*nad5*	371	124	CCA (P) => CTA (L)	0.9
54	*nad5*	395	132	TCT (S) => TTT (F)	0.9
55	*nad5*	503	168	CCT (P) => CTT (L)	1
56	*nad5*	536	179	CCT (P) => CTT (L)	1
57	*nad5*	626	209	TCT (S) => TTT (F)	0.9
58	*nad5*	628	210	CGC (R) => TGC (C)	0.9
59	*nad5*	673	225	CTT (L) => TTT (F)	0.9
60	*nad5*	710	237	TCG (S) => TTG (L)	1
61	*nad5*	722	241	TCA (S) => TTA (L)	1
62	*nad5*	832	278	CCA (P) => TCA (S)	0.9
63	*nad5*	872	291	ACG (T) => ATG (M)	1
64	*nad5*	1307	436	TCA (S) => TTA (L)	1
65	*nad4*	29	10	TCC (S) => TTC (F)	0.67
66	*nad4*	74	25	ACT (T) => ATT (I)	0.89
67	*nad4*	77	26	CCT (P) => CTT (L)	0.78
68	*nad4*	107	36	CCG (P) => CTG (L)	1
69	*nad4*	154	52	CCC (P) => TCC (S)	1
70	*nad4*	158	53	CCT (P) => CTT (L)	1
71	*nad4*	166	56	CGG (R) => TGG (W)	1
72	*nad4*	197	66	TCT (S) => TTT (F)	1
73	*nad4*	362	121	ACA (T) => ATA (I)	0.89
74	*nad4*	368	123	TCT (S) => TTT (F)	1
75	*nad4*	376	126	CGT (R) => TGT (C)	0.78
76	*nad4*	403	135	CGC (R) => TGC (C)	1
77	*nad4*	416	139	CCT (P) => CTT (L)	0.89
78	*nad4*	433	145	CTT (L) => TTT (F)	1
79	*nad4*	436	146	CCC (P) => TTC (F)	0.89
80	*nad4*	437	146	CCC (P) => TTC (F)	0.89
81	*nad4*	449	150	CCA (P) => CTA (L)	1
82	*nad4*	547	183	CTC (L) => TTC (F)	0.67
83	*nad4*	1336	446	CAC (H) => TAC (Y)	1
84	*nad4*	1352	451	CCG (P) => CTG (L)	1
85	*nad4*	1357	453	CGC (R) => TGC (C)	1
86	*atp6*	37	13	CCA (P) => TCA (S)	0.75
87	*atp6*	116	39	TCA (S) => TTA (L)	1
88	*atp6*	167	56	CCG (P) => CTG (L)	1
89	*atp6*	173	58	CCG (P) => CTG (L)	1
90	*atp6*	224	75	TCC (S) => TTC (F)	1
91	*atp6*	229	77	CGC (R) => TGC (C)	0.75
92	*atp6*	236	79	TCG (S) => TTG (L)	0.67
93	*atp6*	254	85	TCG (S) => TTG (L)	1
94	*atp6*	262	88	CGT (R) => TGT (C)	1
95	*atp6*	269	90	CCC (P) => CTC (L)	1
96	*atp6*	401	134	TCA (S) => TTA (L)	1
97	*atp6*	460	154	CCT (P) => TCT (S)	1
98	*atp6*	463	155	CAT (H) => TAT (Y)	1
99	*atp6*	485	162	CCA (P) => CTA (L)	1
100	*atp6*	527	176	TCA (S) => TTA (L)	1
101	*atp6*	548	183	TCC (S) => TTC (F)	1
102	*atp6*	635	212	CCG (P) => CTG (L)	1
103	*atp6*	656	219	TCA (S) => TTA (L)	1
104	*atp6*	664	222	CAT (H) => TAT (Y)	1
105	*atp6*	671	224	TCT (S) => TTT (F)	1
106	*atp6*	680	227	TCA (S) => TTA (L)	1
107	*atp6*	707	236	ACA (T) => ATA (I)	0.92
108	*atp6*	718	240	CAA (Q) => TAA (X)	1
109	*mttB*	58	20	CAT (H) => TAT (Y)	0.88
110	*mttB*	83	28	TCG (S) => TTG (L)	0.88
111	*mttB*	91	31	CCA (P) => TCA (S)	1
112	*mttB*	127	43	CGT (R) => TGT (C)	0.88
113	*mttB*	134	45	CCA (P) => CTA (L)	0.62
114	*mttB*	164	55	TCC (S) => TTC (F)	0.75
115	*mttB*	196	66	CCG (P) => TCG (S)	1
116	*mttB*	253	85	CGT (R) => TGT (C)	0.62
117	*mttB*	290	97	TCT (S) => TTT (F)	1
118	*mttB*	299	100	TCG (S) => TTG (L)	0.75
119	*ccmB*	28	10	CAT (H) => TAT (Y)	0.89
120	*ccmB*	43	15	CCC (P) => TCC (S)	0.67
121	*ccmB*	71	24	CCA (P) => CTA (L)	1
122	*ccmB*	80	27	TCG (S) => TTG (L)	1
123	*ccmB*	128	43	TCA (S) => TTA (L)	1
124	*ccmB*	137	46	TCC (S) => TTC (F)	1
125	*ccmB*	149	50	CCG (P) => CTG (L)	1
126	*ccmB*	154	52	CGG (R) => TGG (W)	1
127	*ccmB*	160	54	CCT (P) => TCT (S)	0.67
128	*ccmB*	164	55	CCG (P) =>=> CTG (L)	0.89
129	*ccmB*	172	58	CCT (P) => TCT (S)	0.89
130	*ccmB*	179	60	CCT (P) => CTT (L)	1
131	*ccmB*	193	65	CCT (P) => TTT (F)	0.89
132	*ccmB*	194	65	CCT (P) => TTT (F)	0.89
133	*ccmB*	286	96	CGG (R) => TGG (W)	1
134	*ccmB*	304	102	CGT (R) => TGT (C)	0.78
135	*ccmB*	313	105	CGT (R) => TGT (C)	0.89
136	*ccmB*	338	113	CCG (P) => CTG (L)	1
137	*ccmB*	367	123	CGG (R) => TGG (W)	0.78
138	*ccmB*	424	142	CGT (R) => TGT (C)	0.89
139	*ccmB*	428	143	TCG (S) => TTG (L)	1
140	*ccmB*	467	156	TCG (S) => TTG (L)	0.89
141	*ccmB*	476	159	CCA (P) => CTA (L)	0.89
142	*ccmB*	485	162	TCA (S) => TTA (L)	1
143	*ccmB*	494	165	TCA (S) => TTA (L)	1
144	*ccmB*	503	168	CCA (P) => CTA (L)	1
145	*ccmB*	512	171	TCT (S) => TTT (F)	1
146	*ccmB*	514	172	CGT (R) => TGT (C)	1
147	*ccmB*	551	184	TCA (S) => TTA (L)	1
148	*ccmB*	554	185	TCG (S) => TTG (L)	0.89
149	*ccmB*	566	189	TCC (S) => TTC (F)	0.78
150	*ccmB*	569	190	TCT (S) => TTT (F)	0.78
151	*ccmB*	572	191	CCG (P) => CTG (L)	1
152	*ccmB*	596	199	TCG (S) => TTG (L)	0.89
153	*rpl10*	101	34	TCG (S) => TTG (L)	0.83
154	*rpl10*	239	80	TCG (S) => TTG (L)	0.83
155	*rpl10*	314	105	TCA (S) => TTA (L)	0.83
156	*rps7*	152	51	CCA (P) => CTA (L)	0.75
157	*rps7*	343	115	CAC (H) => TAC (Y)	0.62
158	*rps7*	368	123	TCA (S) => TTA (L)	0.88
159	*atp1*	1039	347	CCC (P) => TCC (S)	1
160	*atp1*	1064	355	TCG (S) => TTG (L)	1
161	*atp1*	1178	393	TCA (S) => TTA (L)	0.9
162	*atp1*	1216	406	CTT (L) => TTT (F)	1
163	*atp1*	1292	431	CCG (P) => CTG (L)	0.8
164	*atp1*	1415	472	CCA (P) => CTA (L)	1
165	*atp1*	1490	497	CCA (P) => CTA (L)	0.9
166	*atp9*	20	7	TCA (S) => TTA (L)	1
167	*atp9*	50	17	TCA (S) => TTA (L)	1
168	*atp9*	82	28	CTT (L) => TTT (F)	1
169	*atp9*	92	31	TCG (S) => TTG (L)	1
170	*atp9*	134	45	TCA (S) => TTA (L)	1
171	*atp9*	182	61	TCG (S) => TTG (L)	1
172	*atp9*	191	64	CCA (P) => CTA (L)	1
173	*atp9*	212	71	TCA (S) => TTA (L)	1
174	*atp9*	215	72	TCC (S) => TTC (F)	1
175	*atp9*	223	75	CGA (R) => TGA (X)	1
176	*sdh3*	67	23	CCC (P) => TCC (S)	1
177	*sdh3*	376	126	CTC (L) => TTC (F)	0.83
178	*rpl16*	79	27	CAG (Q) => TAG (X)	1
179	*rpl16*	227	76	ACT (T) => ATT (I)	1
180	*rpl16*	355	119	CTC (L) => TTC (F)	0.89
181	*rpl16*	524	175	CCA (P) => CTA (L)	1
182	*rpl16*	530	177	TCG (S) => TTG (L)	0.75
183	*rps3*	314	105	CCA (P) => CTA (L)	0.86
184	*rps3*	647	216	CCG (P) => CTG (L)	1
185	*rps3*	674	225	CCG (P) => CTG (L)	0.86
186	*rps3*	785	262	TCA (S) => TTA (L)	1
187	*rps3*	838	280	CGT (R) => TGT (C)	1
188	*rps3*	902	301	TCA (S) => TTA (L)	0.86
189	*rps19*	62	21	TCG (S) => TTG (L)	1
190	*rps19*	109	37	CCT (P) => TTT (F)	1
191	*rps19*	110	37	CCT (P) => TTT (F)	1
192	*rpl2*	215	72	CCA (P) => CTA (L)	0.75
193	*rpl2*	329	110	CCA (P) => CTA (L)	1
194	*rpl2*	494	165	GCG (A) => GTG (V)	0.67
195	*rpl2*	517	173	CTC (L) => TTC (F)	1
196	*rpl2*	550	184	CCC (P) => TCC (S)	1
197	*atp8*	47	16	TCA (S) => TTA (L)	1
198	*atp8*	58	20	CTC (L) => TTC (F)	1
199	*atp8*	452	151	CCA (P) => CTA (L)	0.75
200	*cox3*	289	97	CTT (L) => TTT (F)	0.92
201	*cox3*	304	102	CGG (R) => TGG (W)	1
202	*cox3*	311	104	TCT (S) => TTT (F)	0.92
203	*cox3*	314	105	TCT (S) => TTT (F)	0.92
204	*cox3*	419	140	CCC (P) => CTC (L)	1
205	*cox3*	422	141	CCT (P) => CTT (L)	0.92
206	*cox3*	512	171	TCA (S) => TTA (L)	0.75
207	*cox3*	653	218	TCG (S) => TTG (L)	1
208	*cox3*	754	252	CGG (R) => TGG (W)	0.92
209	*cox3*	764	255	CCA (P) => CTA (L)	0.92
210	*sdh4*	155	52	CCA (P) => CTA (L)	0.88
211	*sdh4*	203	68	CCA (P) => CTA (L)	0.75
212	*sdh4*	259	87	CAT (H) => TAT (Y)	0.88
213	*cox1*	155	52	TCT (S) => TTT (F)	1
214	*cox1*	167	56	TCT (S) => TTT (F)	1
215	*cox1*	265	89	CCA (P) => TCA (S)	1
216	*cox1*	356	119	TCA (S) => TTA (L)	1
217	*cox1*	365	122	TCT (S) => TTT (F)	1
218	*cox1*	428	143	TCC (S) => TTC (F)	1
219	*cox1*	464	155	TCA (S) => TTA (L)	1
220	*cox1*	503	168	CCA (P) => CTA (L)	1
221	*cox1*	581	194	TCT (S) => TTT (F)	1
222	*cox1*	628	210	CGG (R) => TGG (W)	1
223	*cox1*	659	220	CCC (P) => CTC (L)	1
224	*cox1*	674	225	TCC (S) => TTC (F)	1
225	*cox1*	758	253	ACA (T) => ATA (I)	1
226	*cox1*	773	258	TCT (S) => TTT (F)	1
227	*cox1*	950	317	TCC (S) => TTC (F)	1
228	*cox1*	1099	367	CAC (H) => TAC (Y)	1
229	*cox1*	1187	396	CCG (P) => CTG (L)	0.89
230	*cox1*	1318	440	CGT (R) => TGT (C)	0.78
231	*cox1*	1346	449	TCA (S) => TTA (L)	1
232	*cox1*	1402	468	CCA (P) => TCA (S)	1
233	*cox1*	1412	471	TCG (S) => TTG (L)	1
234	*nad7*	38	13	TCG (S) => TTG (L)	0.75
235	*nad7*	77	26	TCA (S) => TTA (L)	1
236	*nad7*	83	28	TCA (S) => TTA (L)	1
237	*nad7*	137	46	TCA (S) => TTA (L)	1
238	*nad7*	205	69	CAT (H) => TAT (Y)	1
239	*nad7*	212	71	TCA (S) => TTA (L)	1
240	*nad7*	277	93	CGT (R) => TGT (C)	1
241	*nad7*	296	99	TCA (S) => TTA (L)	0.88
242	*nad7*	305	102	TCA (S) => TTA (L)	1
243	*nad7*	344	115	TCA (S) => TTA (L)	1
244	*nad7*	494	165	TCC (S) => TTC (F)	1
245	*nad7*	539	180	TCA (S) => TTA (L)	0.88
246	*nad7*	812	271	TCA (S) => TTA (L)	0.88
247	*nad7*	859	287	CCT (P) => TCT (S)	0.88
248	*nad7*	943	315	CGT (R) => TGT (C)	1
249	*nad7*	965	322	TCT (S) => TTT (F)	1
250	*nad7*	989	330	TCT (S) => TTT (F)	1
251	*nad7*	1010	337	CCA (P) => CTA (L)	1
252	*nad7*	1052	351	TCT (S) => TTT (F)	1
253	*nad9*	428	143	TCC (S) => TTC (F)	0.73
254	*nad9*	506	169	TCT (S) => TTT (F)	0.75
255	*nad9*	527	176	CCA (P) => CTA (L)	0.92
256	*nad9*	581	194	TCG (S) => TTG (L)	0.92
257	*nad9*	604	202	CAT (H) => TAT (Y)	1
258	*nad9*	712	238	CCG (P) => TCG (S)	0.83
259	*nad9*	742	248	CGG (R) => TGG (W)	1
260	*nad9*	782	261	TCC (S) => TTC (F)	1
261	*nad9*	812	271	TCA (S) => TTA (L)	1
262	*nad9*	853	285	CTT (L) => TTT (F)	1
263	*nad9*	953	318	TCT (S) => TTT (F)	1
264	*nad4L*	11	4	TCT (S) => TTT (F)	1
265	*nad4L*	17	6	TCA (S) => TTA (L)	1
266	*nad4L*	25	9	CGG (R) => TGG (W)	1
267	*nad4L*	56	19	CCT (P) => CTT (L)	1
268	*nad4L*	65	22	TCA (S) => TTA (L)	1
269	*nad4L*	70	24	CCA (P) => TCA (S)	1
270	*nad4L*	80	27	TCA (S) => TTA (L)	1
271	*nad4L*	101	34	TCG (S) => TTG (L)	0.88
272	*nad4L*	128	43	TCG (S) => TTG (L)	1
273	*nad4L*	149	50	TCA (S) => TTA (L)	0.75
274	*nad4L*	158	53	TCA (S) => TTA (L)	0.88
275	*nad4L*	167	56	CCA (P) => CTA (L)	0.88
276	*nad4L*	200	67	TCA (S) => TTA (L)	1
277	*nad4L*	251	84	TCT (S) => TTT (F)	0.88
278	*atp4*	71	24	TCA (S) => TTA (L)	1
279	*atp4*	89	30	TCA (S) => TTA (L)	1
280	*atp4*	118	40	CGT (R) => TGT (C)	0.71
281	*atp4*	215	72	TCG (S) => TTG (L)	1
282	*atp4*	248	83	CCT (P) => CTT (L)	1
283	*atp4*	395	132	TCA (S) => TTA (L)	1
284	*atp4*	407	136	CCA (P) => CTA (L)	0.71
285	*atp4*	416	139	ACT (T) => ATT (I)	0.86
286	*ccmC*	76	26	CGG (R) => TGG (W)	0.78
287	*ccmC*	103	35	CAT (H) => TAT (Y)	1
288	*ccmC*	115	39	CGG (R) => TGG (W)	0.78
289	*ccmC*	133	45	CTT (L) => TTT (F)	0.67
290	*ccmC*	161	54	CCG (P) => CTG (L)	0.78
291	*ccmC*	179	60	GCG (A) => GTG (V)	0.78
292	*ccmC*	184	62	CGG (R) => TGG (W)	1
293	*ccmC*	299	100	TCT (S) => TTT (F)	1
294	*ccmC*	331	111	CGG (R) => TGG (W)	1
295	*ccmC*	395	132	TCG (S) => TTG (L)	1
296	*ccmC*	400	134	CTT (L) => TTT (F)	0.89
297	*ccmC*	421	141	CGT (R) => TGT (C)	0.78
298	*ccmC*	436	146	CCT (P) => TCT (S)	0.89
299	*ccmC*	446	149	CCG (P) => CTG (L)	0.78
300	*ccmC*	451	151	CCT (P) => TCT (S)	1
301	*ccmC*	458	153	TCA (S) => TTA (L)	0.78
302	*ccmC*	463	155	CGT (R) => TGT (C)	1
303	*ccmC*	467	156	GCT (A) => GTT (V)	0.78
304	*ccmC*	473	158	CCG (P) => CTG (L)	1
305	*ccmC*	497	166	TCT (S) => TTT (F)	1
306	*ccmC*	521	174	TCG (S) => TTG (L)	1
307	*ccmC*	548	183	TCT (S) => TTT (F)	1
308	*ccmC*	568	190	CCT (P) => TCT (S)	1
309	*ccmC*	575	192	CCC (P) => CTC (L)	1
310	*ccmC*	605	202	TCC (S) => TTC (F)	1
311	*ccmC*	608	203	CCC (P) => CTC (L)	0.89
312	*ccmC*	614	205	TCA (S) => TTA (L)	0.78
313	*ccmC*	619	207	CGT (R) => TGT (C)	0.78
314	*ccmC*	650	217	CCT (P) => CTT (L)	0.78
315	*ccmC*	656	219	CCA (P) => CTA (L)	0.89
316	*ccmC*	673	225	CCT (P) => TCT (S)	0.78
317	*cox2*	71	24	TCT (S) => TTT (F)	1
318	*cox2*	161	54	TCA (S) => TTA (L)	0.95
319	*cox2*	163	55	CGG (R) => TGG (W)	1
320	*cox2*	253	85	CGG (R) => TGG (W)	1
321	*cox2*	278	93	CCG (P) => CTG (L)	1
322	*cox2*	379	127	CGG (R) => TGG (W)	1
323	*cox2*	443	148	ACG (T) => ATG (M)	1
324	*cox2*	461	154	CCA (P) => CTA (L)	1
325	*cox2*	476	159	TCA (S) => TTA (L)	1
326	*cox2*	544	182	CCT (P) => TCT (S)	1
327	*cox2*	557	186	CCT (P) => CTT (L)	1
328	*cox2*	581	194	TCA (S) => TTA (L)	1
329	*cox2*	632	211	TCG (S) => TTG (L)	0.84
330	*cox2*	698	233	ACG (T) => ATG (M)	1
331	*cox2*	742	248	CGG (R) => TGG (W)	1
332	*rps13*	5	2	TCA (S) => TTA (L)	0.6
333	*rps13*	26	9	TCA (S) => TTA (L)	0.9
334	*rps13*	56	19	TCA (S) => TTA (L)	0.9
335	*rps13*	100	34	CGT (R) => TGT (C)	0.9
336	*rps13*	287	96	TCG (S) => TTG (L)	1
337	*rps4*	133	45	CCG (P) => TCG (S)	0.67
338	*rps4*	164	55	TCA (S) => TTA (L)	1
339	*rps4*	184	62	CCC (P) => TCC (S)	0.83
340	*rps4*	193	65	CAT (H) => TAT (Y)	1
341	*rps4*	257	86	CCA (P) => CTA (L)	1
342	*rps4*	266	89	CCA (P) => CTA (L)	0.83
343	*rps4*	278	93	TCG (S) => TTG (L)	0.67
344	*rps4*	290	97	CCG (P) => CTG (L)	0.83
345	*rps4*	335	112	CCG (P) => CTG (L)	1
346	*rps4*	482	161	TCA (S) => TTA (L)	1
347	*rps4*	914	305	TCG (S) => TTG (L)	0.83
348	*rps4*	925	309	CAT (H) => TAT (Y)	0.83
349	*rps4*	935	312	CCA (P) => CTA (L)	0.67
350	*rps4*	950	317	TCT (S) => TTT (F)	1
351	*rps4*	1001	334	CCA (P) => CTA (L)	0.83
352	*rps4*	1010	337	CCT (P) => CTT (L)	1
353	*rps4*	1015	339	CGG (R) => TGG (W)	1
354	*nad1*	8	3	CCT (P) => CTT (L)	0.9
355	*nad1*	65	22	TCC (S) => TTC (F)	1
356	*nad1*	100	34	CCT (P) => TCT (S)	0.9
357	*nad1*	149	50	GCG (A) => GTG (V)	0.9
358	*nad1*	209	70	TCC (S) => TTC (F)	1
359	*nad1*	308	103	TCA (S) => TTA (L)	1
360	*nad1*	434	145	ACT (T) => ATT (I)	1
361	*nad6*	7	3	CTT (L) => TTT (F)	1
362	*nad6*	83	28	TCG (S) => TTG (L)	1
363	*nad6*	88	30	CCC (P) => TTC (F)	0.7
364	*nad6*	89	30	CCC (P) => TTC (F)	0.7
365	*nad6*	95	32	CCA (P) => CTA (L)	1
366	*nad6*	103	35	CGC (R) => TGC (C)	1
367	*nad6*	161	54	CCA (P) => CTA (L)	1
368	*nad6*	169	57	CAT (H) => TAT (Y)	1
369	*nad6*	191	64	TCA (S) => TTA (L)	1
370	*nad6*	446	149	TCC (S) => TTC (F)	1
371	*nad6*	463	155	CCT (P) => TCT (S)	0.8
372	*nad6*	569	190	TCT (S) => TTT (F)	1
373	*nad2*	26	9	TCC (S) => TTC (F)	0.89
374	*nad2*	203	68	TCT (S) => TTT (F)	0.67
375	*nad2*	206	69	TCC (S) => TTC (F)	1
376	*nad2*	230	77	TCT (S) => TTT (F)	1
377	*nad2*	236	79	TCC (S) => TTC (F)	0.67
378	*nad2*	251	84	CCA (P) => CTA (L)	1
379	*nad2*	262	88	CGC (R) => TGC (C)	1
380	*nad2*	289	97	CAT (H) => TAT (Y)	1
381	*nad2*	296	99	TCA (S) => TTA (L)	1
382	*nad2*	323	108	CCT (P) => CTT (L)	1
383	*nad2*	392	131	TCG (S) => TTG (L)	1
384	*rps12*	71	24	TCG (S) => TTG (L)	0.94
385	*rps12*	100	34	CGC (R) => TGC (C)	1
386	*rps12*	104	35	CCG (P) => CTG (L)	1
387	*rps12*	196	66	CAC (H) => TAC (Y)	0.94
388	*rps12*	221	74	TCG (S) => TTG (L)	0.88
389	*rps12*	269	90	TCG (S) => TTG (L)	0.94
390	*rps12*	284	95	TCC (S) => TTC (F)	0.76
391	*nad3*	5	2	TCA (S) => TTA (L)	0.79
392	*nad3*	44	15	CCG (P) => CTG (L)	1
393	*nad3*	62	21	CCA (P) => CTA (L)	0.95
394	*nad3*	80	27	CCA (P) => CTA (L)	1
395	*nad3*	146	49	TCC (S) => TTC (F)	1
396	*nad3*	208	70	CCT (P) => TTT (F)	0.95
397	*nad3*	209	70	CCT (P) => TTT (F)	0.95
398	*nad3*	215	72	CCG (P) => CTG (L)	1
399	*nad3*	230	77	TCC (S) => TTC (F)	0.86
400	*nad3*	247	83	CCT (P) => TCT (S)	1
401	*nad3*	251	84	CCC (P) => CTC (L)	0.91
402	*nad3*	266	89	CCG (P) => CTG (L)	1
403	*nad3*	275	92	TCT (S) => TTT (F)	1
404	*nad3*	317	106	TCT (S) => TTT (F)	0.95
405	*nad3*	344	115	TCG (S) => TTG (L)	1
406	*nad3*	349	117	CGG (R) => TGG (W)	1
407	*rps1*	23	8	CCT (P) => CTT (L)	0.67
408	*rps1*	56	19	CCT (P) => CTT (L)	0.67
409	*rps1*	380	127	TCA (S) => TTA (L)	0.67

*The cutoff score (C-value) was set to 0.6.

**Online-only Table 5:** Predicted RNA-editing sites in Scaffold 2 of the *C*. *sinensis* var. *assamica* mt genome.

No.	Gene	Nucleotide Position	AA Pos	Effect	Score*
1	*rps19*	116	39	TCG (S) => TTG (L)	1
2	*rps19*	163	55	CCT (P) => TTT (F)	1
3	*rps19*	164	55	CCT (P) => TTT (F)	1
4	*atp9*	53	18	TCA (S) => TTA (L)	1
5	*atp9*	83	28	TCA (S) => TTA (L)	1
6	*cob*	118	40	CCG (P) => TCG (S)	0.92
7	*cob*	178	60	CAC (H) => TAC (Y)	1
8	*cob*	286	96	CTC (L) => TTC (F)	1
9	*cob*	298	100	CAC (H) => TAC (Y)	1
10	*cob*	325	109	CAT (H) => TAT (Y)	1
11	*cob*	358	120	CGG (R) => TGG (W)	1
12	*cob*	419	140	CCA (P) => CTA (L)	1
13	*cob*	568	190	CAT (H) => TAT (Y)	0.92
14	*cob*	680	227	TCT (S) => TTT (F)	1
15	*cob*	808	270	CCC (P) => TCC (S)	1
16	*cob*	853	285	CAT (H) => TAT (Y)	1
17	*cob*	908	303	CCA (P) => CTA (L)	1
18	*cob*	914	305	TCT (S) => TTT (F)	1
19	*cob*	982	328	CAC (H) => TAC (Y)	0.85
20	*cob*	1015	339	CGC (R) => TGC (C)	1
21	*cob*	1084	362	CCT (P) => TCT (S)	1
22	*cob*	1124	375	CCG (P) => CTG (L)	1
23	*rps14*	47	16	GCG (A) => GTG (V)	0.6
24	*rps14*	271	91	CCT (P) => TCT (S)	0.6
25	*rpl5*	35	12	TCA (S) => TTA (L)	0.78
26	*rpl5*	47	16	CCG (P) => CTG (L)	1
27	*rpl5*	59	20	CCG (P) => CTG (L)	0.89
28	*rpl5*	64	22	CAC (H) => TAC (Y)	1
29	*rpl5*	92	31	TCG (S) => TTG (L)	1
30	*rpl5*	172	58	CGC (R) => TGC (C)	0.89
31	*rpl5*	518	173	CCA (P) => CTA (L)	0.89
32	*rpl5*	521	174	CCG (P) => CTG (L)	1
33	*nad2*	110	37	TCT (S) => TTT (F)	1
34	*nad2*	125	42	TCC (S) => TTC (F)	1
35	*nad2*	272	91	TCT (S) => TTT (F)	0.67
36	*nad2*	284	95	TCA (S) => TTA (L)	1
37	*nad2*	293	98	TCT (S) => TTT (F)	1
38	*nad2*	412	138	CAT (H) => TAT (Y)	1
39	*nad2*	442	148	CGT (R) => TGT (C)	0.78
40	*nad2*	446	149	ACT (T) => ATT (I)	1
41	*nad2*	512	171	TCA (S) => TTA (L)	0.78
42	*nad2*	542	181	TCA (S) => TTA (L)	1
43	*nad2*	611	204	TCG (S) => TTG (L)	1
44	*nad2*	731	244	CCA (P) => CTA (L)	0.67
45	*nad2*	760	254	CGT (R) => TGT (C)	1
46	*nad2*	932	311	TCA (S) => TTA (L)	0.67
47	*nad2*	941	314	CCA (P) => CTA (L)	1
48	*nad2*	989	330	TCA (S) => TTA (L)	1
49	*sdh3*	67	23	CCA (P) => TCA (S)	1
50	*sdh3*	74	25	TCC (S) => TTC (F)	1
51	*ccmFc*	38	13	TCC (S) => TTC (F)	0.83
52	*ccmFc*	50	17	CCT (P) => CTT (L)	1
53	*ccmFc*	52	18	CGT (R) => TGT (C)	1
54	*ccmFc*	103	35	CCC (P) => TCC (S)	1
55	*ccmFc*	119	40	TCT (S) => TTT (F)	1
56	*ccmFc*	122	41	TCC (S) => TTC (F)	1
57	*ccmFc*	146	49	CCT (P) => CTT (L)	1
58	*ccmFc*	151	51	CCT (P) => TCT (S)	0.83
59	*ccmFc*	155	52	TCA (S) => TTA (L)	1
60	*ccmFc*	160	54	CCT (P) => TCT (S)	0.67
61	*ccmFc*	203	68	ACG (T) => ATG (M)	1
62	*ccmFc*	305	102	TCA (S) => TTA (L)	0.83
63	*ccmFc*	391	131	CGT (R) => TGT (C)	1
64	*ccmFc*	406	136	CGT (R) => TGT (C)	0.83
65	*ccmFc*	620	207	GCG (A) => GTG (V)	1
66	*ccmFc*	704	235	GCT (A) => GTT (V)	0.83
67	*ccmFc*	1100	367	CCA (P) => CTA (L)	1
68	*ccmFc*	1121	374	TCG (S) => TTG (L)	1
69	*ccmFc*	1276	426	CGA (R) => TGA (X)	1

*The cutoff score (C-value) was set to 0.6.
